# Amplification of pressure waves in laser-assisted endodontics with synchronized delivery of Er:YAG laser pulses

**DOI:** 10.1007/s10103-017-2435-z

**Published:** 2018-01-11

**Authors:** Nejc Lukač, Matija Jezeršek

**Affiliations:** Faculty of Mechanical Engineering, University of Ljubljana, Aškerčeva 6, 1000 Ljubljana, Slovenia

**Keywords:** Endodontics, Er:YAG, Laser-beam deflection probe, Shock wave

## Abstract

When attempting to clean surfaces of dental root canals with laser-induced cavitation bubbles, the resulting cavitation oscillations are significantly prolonged due to friction on the cavity walls and other factors. Consequently, the collapses are less intense and the shock waves that are usually emitted following a bubble’s collapse are diminished or not present at all. A new technique of synchronized laser-pulse delivery intended to enhance the emission of shock waves from collapsed bubbles in fluid-filled endodontic canals is reported. A laser beam deflection probe, a high-speed camera, and shadow photography were used to characterize the induced photoacoustic phenomena during synchronized delivery of Er:YAG laser pulses in a confined volume of water. A shock wave enhancing technique was employed which consists of delivering a second laser pulse at a delay with regard to the first cavitation bubble-forming laser pulse. Influence of the delay between the first and second laser pulses on the generation of pressure and shock waves during the first bubble’s collapse was measured for different laser pulse energies and cavity volumes. Results show that the optimal delay between the two laser pulses is strongly correlated with the cavitation bubble’s oscillation period. Under optimal synchronization conditions, the growth of the second cavitation bubble was observed to accelerate the collapse of the first cavitation bubble, leading to a violent collapse, during which shock waves are emitted. Additionally, shock waves created by the accelerated collapse of the primary cavitation bubble and as well of the accompanying smaller secondary bubbles near the cavity walls were observed. The reported phenomena may have applications in improved laser cleaning of surfaces during laser-assisted dental root canal treatments.

## Introduction

Laser-induced cavitation bubbles have already been proposed for surface cleaning [1]. The cleaning of surfaces is carried out by fluid flow generated when bubbles expand and collapse close to boundaries [2].

An example of the use of laser-induced cavitation bubbles is the laser activated irrigation (LAI) during the dental root canal therapy, using an erbium laser (2940 or 2780 nm) [3–6]. The treatment is based on the delivery of erbium laser pulses into the liquid-filled canal through a fiber tip. The erbium laser light is highly absorptive in water (approximately 1–3 μm penetration depth) [7], which leads to explosive boiling that induces cavitation bubbles.

Photon-induced photoacoustic streaming (PIPS™) is the latest application of LAI, which uses the Er:YAG (2940 nm) laser equipped with a conical and stripped fiber tip [8–14]. With the PIPS technique, the fiber tip is held in the coronal aspect of the access preparation, and very short bursts of very low laser energy are directed down into the canal to stream irrigants throughout the entire root canal system. This technique results in much deeper irrigation than traditional methods (syringe, ultrasonic needle) [9–13], being capable of reaching lateral canals and other outlying structures also in the apical part of the root canal [7, 14], with the major cleaning mechanism being attributed to the liquid vorticity resulting from the laser-induced oscillations of the cavitation bubbles [15, 16].

Also of major concern in root canal irrigation is the effective removal of the biofilm and of the smear layer, which is produced during root canal instrumentation and consists of inorganic and organic material including bacteria and their by-products [17–20]. When LAI was first introduced it was believed that shock waves generated during the bubbles’ collapse would contribute to the efficacy of debridement and removal of the biofilm and organic tissue remains [18, 21]. However, as opposed to within infinite liquid reservoirs, shock waves are considerably diminished or are not present at all when bubbles are created in confined reservoirs such as dental root canals [7, 15]. This is because in confined liquid cavities, the resulting cavitation oscillations are significantly prolonged due to friction on the cavity walls and other factors. Consequently, the collapses are not intense enough to generate shock waves. Current procedures thus still rely on the use of ethylenediaminetetraacetic acid (EDTA) and sodium hypochlorite solutions and are only partially effective in removing the smear layer and biofilm [18–22]. Therefore, further optimization of laser-assisted irrigation and cleaning procedures is called for.

Recently, a synchronized delivery of laser pulses was studied in an infinite liquid reservoir, showing that a resonance effect can be achieved by applying a second laser pulse shortly after the collapse of the primary cavitation bubble to increase the mechanical energy of the secondary oscillation [23]. However, these results have limited value for endodontic applications, as the oscillations of cavitation bubbles in the confined geometry of the root canal vary significantly from the infinite liquid reservoir scenario. In confined reservoirs, secondary oscillations are diminished or not present at all and the collapses happen 2–3 mm below the fiber tip. Therefore, subsequent laser pulses lead to the generation of new cavitation bubbles, physically separate from the primary bubble, and the resonance effect does not take place.

In this paper, we report on a new SWEEPS (shock wave-enhanced emission photoacoustic streaming) technique of synchronized laser-pulse delivery intended to enhance shock waves emitted by collapsed bubbles in confined spaces such as root canals. As the collapse of the laser-induced cavitation bubble is initiated, a second pulse is delivered into the liquid, forming a second cavitation bubble. The growth of the second cavitation bubble accelerates the collapse of the first cavitation bubble, leading to a violent collapse, during which shock waves are emitted. Furthermore, shock waves are also emitted from the collapsing secondary cavitation bubbles that form naturally throughout the entire length of the canal during laser-induced irrigation. Unlike the main cavitation bubbles, the secondary bubbles are in close proximity to canal walls during their collapses, generating shear flows that are able to remove particles from the surface [1]. Additionally, because of their proximity to the canal walls, the emitted shock waves are still propagating at super-sonic speeds as they reach the smear layer, potentially increasing the cleaning mechanism even further. The proposed SWEEPS technique shares similarities with extracorporeal shock wave lithotripsy (ESWL), where focused ultrasonic waves are used to break kidney stones into smaller pieces [24, 25].

## Materials and methods

The cavitation bubbles and the corresponding pressure waves were generated with an Er:YAG laser (LightWalker ATS, Fotona d.o.o, *λ* = 2.94 μm) fitted with an articulated arm and a fiber tip handpiece (H14, Fotona d.o.o). Laser pulses were delivered into liquid-filled canals through fiber tips (flat Fotona VARIAN 600 fiber tip or conical Fotona PIPS 600 fiber) with 600 μm fiber diameter. Although both types of fiber tips were tried in most of the experiments, the presented data is mainly for the experiments obtained with the flat fiber tip. This is because under the SWEEPS conditions the cone of the conical fiber tip became very quickly damaged, making the collected data unreliable. We attribute this observation to the significant amplification of the pressure waves in the vicinity of the accelerated collapse of the first bubble under the SWEEPS conditions.

The induced photoacoustic phenomena in the confined liquid space were characterized with two experimental setups. One of the setups was a laser beam deflection probe (LBDP), which measured the amplitudes of pressure waves based on changes in the refractive index gradient at a single point with high temporal resolution [26]. The other setup involved high-speed camera acquisition and shadow photography used to visualize cavitation bubbles and the emission of resulting shock waves. Bubble oscillation periods and volumes were determined from the captured sequences of images.

### Measurement of pressure waves using laser beam deflection probe

**Fig. 1:**
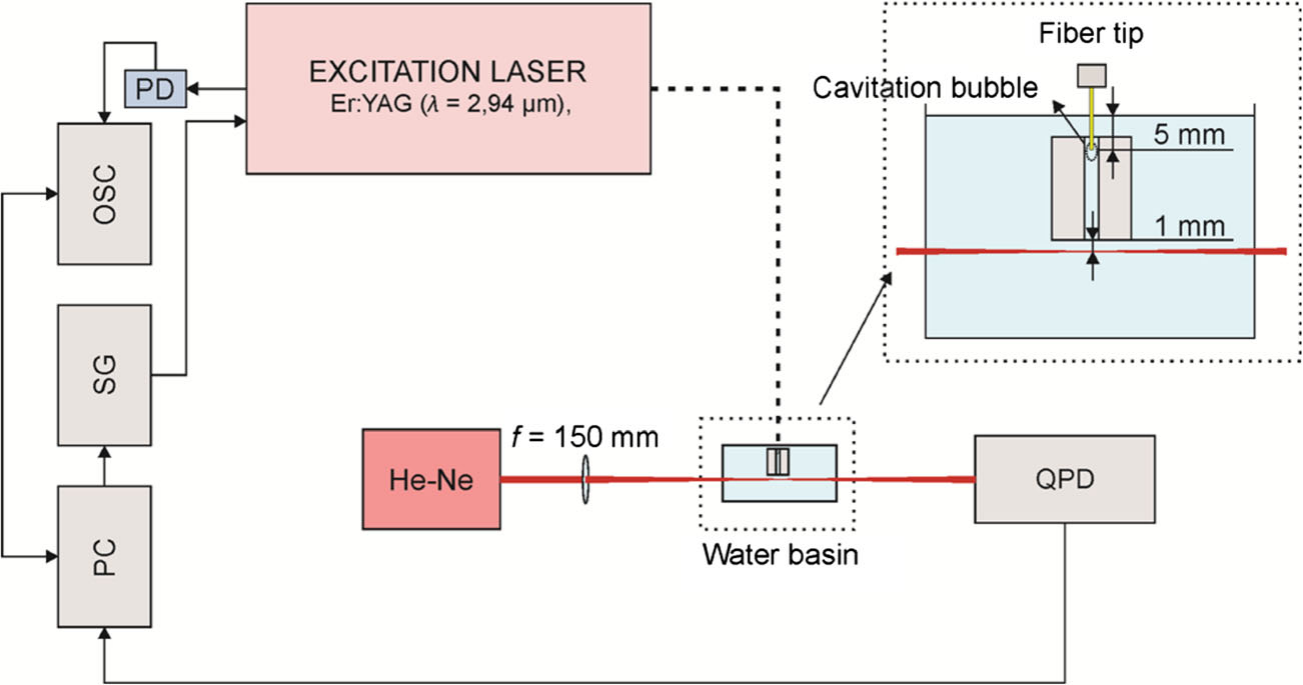
A schematic overview of the experimental setup for laser beam deflection probe measurements. A block of aluminum with canals of different diameters was submerged 3 mm deep in a basin of distilled water. A flat fiber positioned in the center of the cross section of the canal, 5 mm below the water surface, was used to deliver the Er:YAG laser pulses with pulse energy *E*_p_ = 20 mJ and pulse width *t*_p_ = 50 μs. A signal generator (SG) controlled by a personal computer (PC) was used to trigger the excitation lasers. A photodiode (PD) was used to detect the Er:YAG laser pulses. The measuring system consisted of a He-Ne laser beam (*λ* = 633 nm) focused to a measuring spot 1 mm below the lower edge of the canal and centered on a quadrant photodiode (QPD). Signals from the QDP were recorded using an oscilloscope (OSC)

The experimental setup for measuring the amplitudes of pressure waves is shown schematically in Fig. 1. A block of aluminum with *L* = 25 mm long open-ended canals of different diameters (2, 3, 6, and 8 mm) was submerged 3 mm deep in a basin of distilled water (100 × 100 × 70 mm). A flat fiber tip (VARIAN 600), positioned in the center of the cross section of the canal, 5 mm below the water surface, was used to deliver the excitation laser pulses.

A signal generator controlled by a personal computer was used to trigger the excitation laser. A 60-MHz InAs photodiode was used to detect and characterize the temporal profiles of the Er:YAG laser pulses.

The laser beam deflection probe consisted of a He-Ne laser beam (*λ* = 633 nm) focused to a measuring spot 1 mm below the lower edge of the canal (26 mm below the fiber tip) and centered on a quadrant photodiode (QPD). The refractive index gradient produced by the propagation of a pressure wave through the water caused the deflection of the probe laser beam and a change in the signal of the QPD. Because the probe laser beam was positioned directly below the source of the pressure wave, only the vertical deflection was measured by subtracting the sum of signals from the upper two quadrants from the sum of signals from the lower two quadrants of the photodiode.

**Fig. 2:**
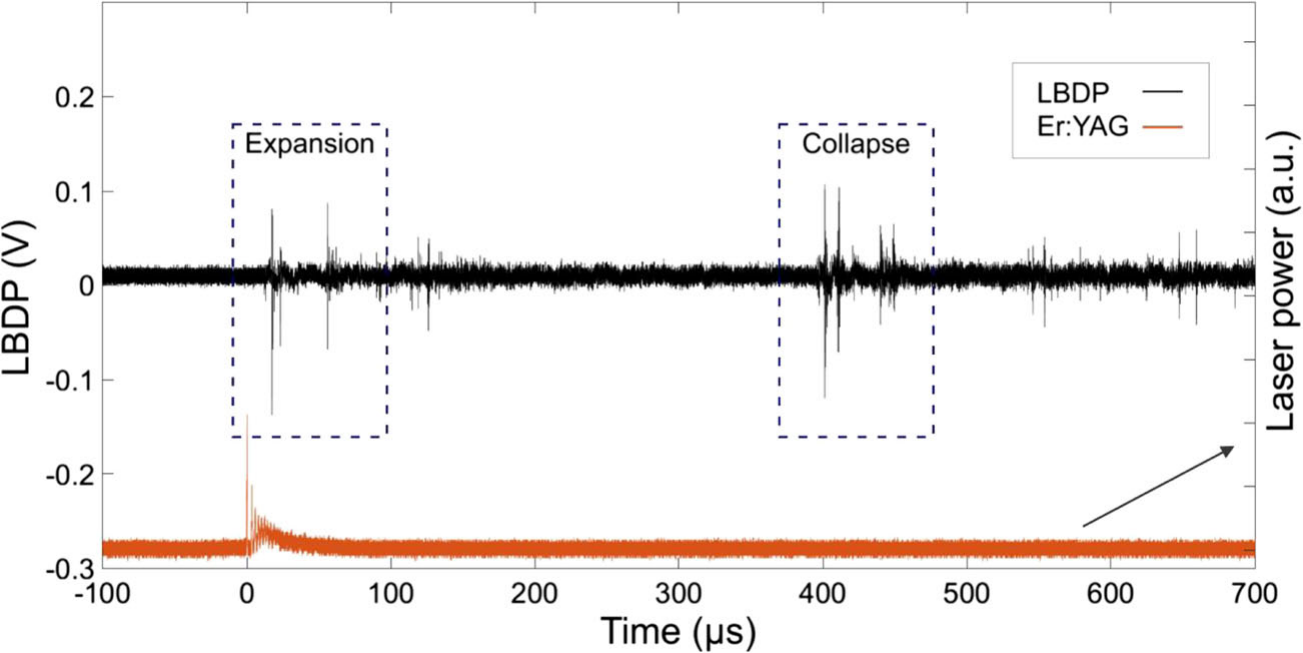
Typical signal from the LBDP (upper black signal) following a single Er:YAG laser pulse (lower red signal) with pulse energy *E*_p_ = 20 mJ and pulse width *t*_p_ = 50 μs, delivered through a flat fiber tip in a 6-mm-diameter canal. The two marked regions represent the expansion and collapse phase of the laser-induced oscillation bubble

Figure 2 shows a typical LBDP signal (black line) produced by the propagation of a pressure wave following a single laser pulse with energy *E*_p_ = 20 mJ and pulse width *t*_p_ = 50 μs. The temporal profile of the laser pulse is represented by the red line on the same graph. Two particular regions of interest are distinguishable from the LBDP signal: the first is the result of the rapid expansion of the laser-induced oscillation bubble (see the dashed rectangle on the left side of Fig. 2), and the second is the result of the oscillation bubble’s collapse (see the dashed rectangle on the right side of Fig. 2). The first peak in the LBDP signal at expansion corresponds to the direct pressure wave, while the second peak (approximately 40 us after the first one) is the reflection from the bottom of the water reservoir. The same pair of peaks can be seen during the collapse.

**Fig. 3:**
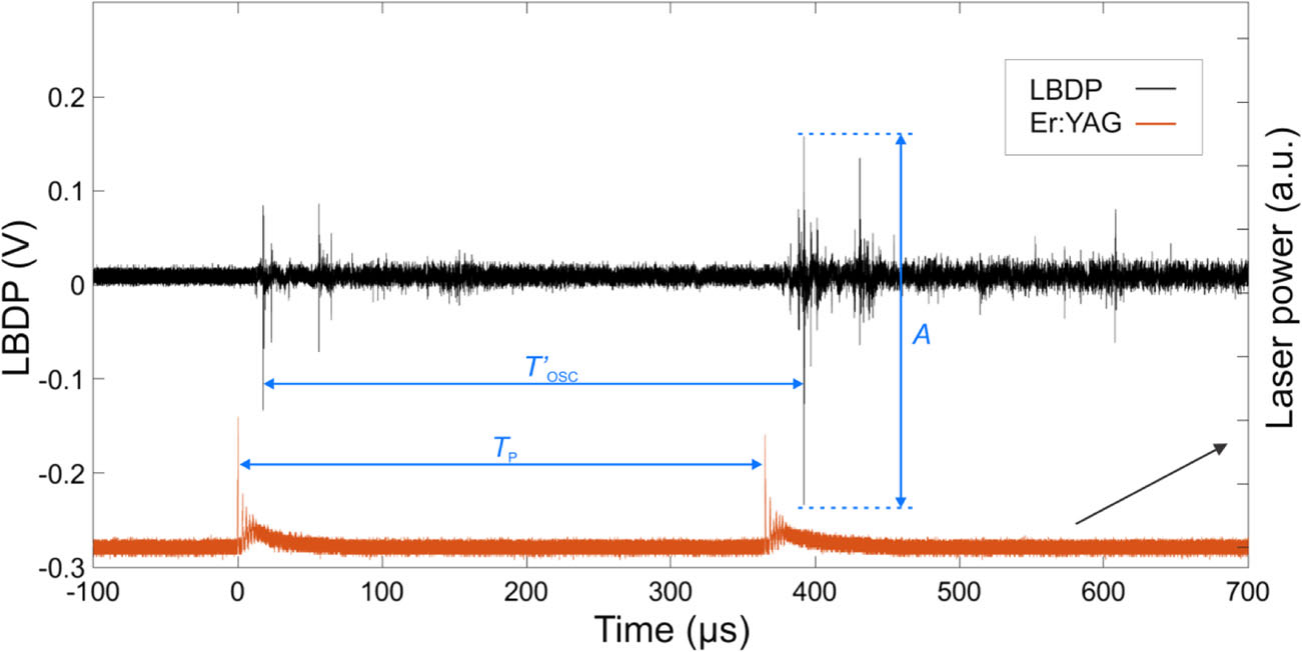
Typical signal from the LBDP (upper black signal) following two Er:YAG laser pulses with pulse energy *E*_p_ = 20 mJ and pulse width *t*_p_ = 50 μs (lower red signal) separated by a delay (*T*_p_), delivered through a flat fiber tip, in a 6-mm-diameter canal. The peak-to-peak amplitude (*A*) of the LBDP signal at the time of the oscillation bubble’s collapse was measured. The oscillation period of the bubble, measured by the LBDP, is denoted as *T*′_OSC_

Figure 3 shows a typical LBDP signal when two individual laser pulses (*E*_p_ = 20 mJ and pulse width *t*_p_ = 50 μs for each pulse) were delivered into the liquid, separated by a temporal delay *T*_p_. In the particular case shown in Fig. 3, the second laser pulse was delivered at a time just before the collapse of the first cavitation bubble. Under such conditions, the collapse of the first bubble is accelerated, which leads to a more intense pressure wave generation in comparison to when a second laser pulse is absent (see Fig. 2). This amplification was characterized by measuring the collapse amplitude (*A*), defined as the peak-to-peak amplitude of the LBDP signal during the first bubble’s collapse phase. The temporal separation (the LBDP oscillation time; *T*′_OSC_) between the ‘expansion’ and ‘collapse’ LBDP signals was also measured. Note that the LBDP oscillation time *T*′_OSC_ corresponds only approximately to the actual oscillation period of the bubble since the temporal separation between the LBDP signals can be affected by the spatial movement of the oscillating bubble relative to the LBDP probe (and by other factors further discussed below).

In order to find the optimal delay (*T*_p_^*^) where the collapse amplitude is maximal (A^*^), a series of measurements was conducted by varying *T*_p_ in a range from 200 to 800 μs in 1 μs intervals and recording *A* and *T*′_OSC_ for different canal diameters (2, 3, 6, and 8 mm).

### High-speed camera and shadow photography

Two additional experimental systems were used to record the generated shock waves during the synchronized delivery of laser pulses and to measure the dependence of the bubble’s oscillation period (*T*_OSC_) on the laser pulse energy, cavity diameter, and fiber tip position.

The shock waves were recorded with a shadow-graphic setup using 30 ps long frequency-doubled Nd:YAG (*λ* = 532 nm) illumination pulses (Ekspla, Lithuania, PL2250-SH-TH), imaged through a microscope by a charge-coupled device (CCD) camera (Basler AG, Germany, scA1400-17 fm, 1.4 Mpx). The experimental system is basically the same as described in ref. [15].

**Fig. 4:**
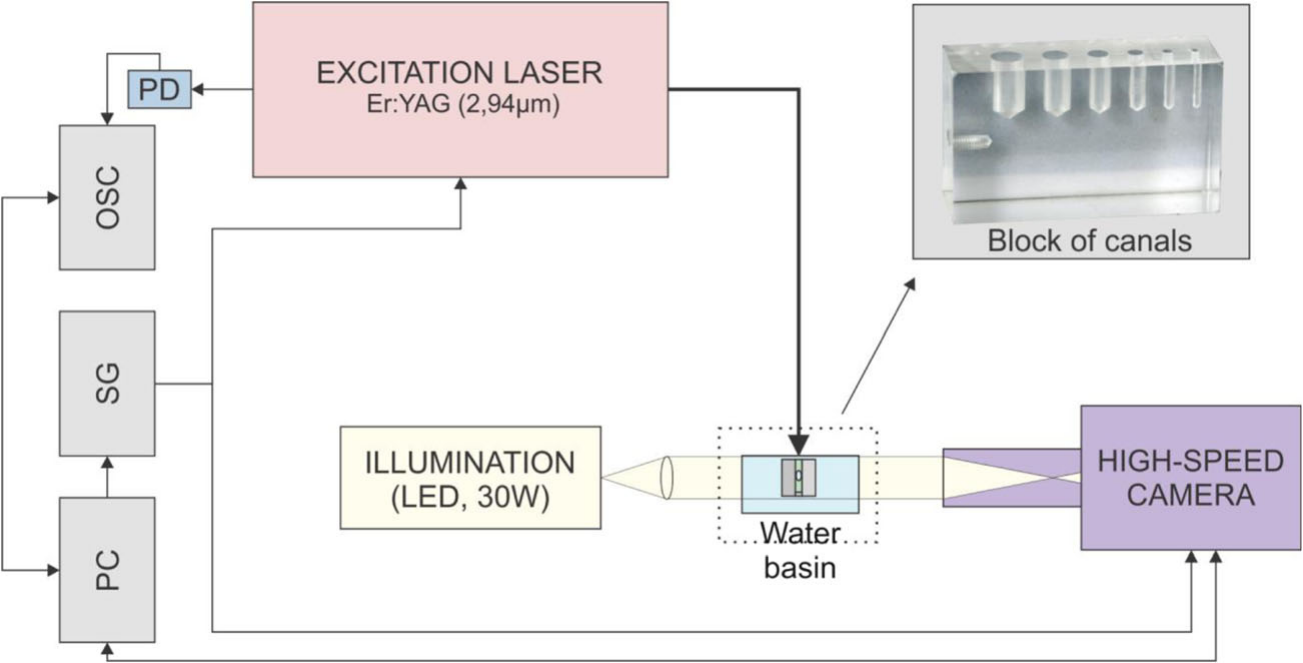
Experimental system for measuring the dependence of the cavitation bubble’s oscillation period (*T*_osc_) on different parameters (laser pulse energy, cavity diameter, and fiber tip position). A block of plexiglass with canals of varying diameters (1.5–6 mm) and lengths (10 and 20 mm closed-ended and 30 mm open-ended canals) was used to simulate various cavity dimensions. The block was submerged 3 mm deep in a basin of distilled water and a conical fiber tip was positioned in the center of the cross section of the canal, 5 mm below the water surface, to deliver the Er:YAG laser pulses with pulse energies ranging from 5 to 30 mJ and pulse width *t*_p_ = 50 μs. A signal generator (SG) was used to trigger the excitation laser and the camera (Photron Fastcam SA-Z)

Figure 4 shows the experimental system for measuring the dependence of the cavitation bubble’s oscillation period (*T*osc) on different parameters (laser pulse energy, cavity diameter, and fiber tip position). A block of acrylic glass with canals of varying diameters (1.5–6 mm) and lengths (10 mm and 20 mm closed-ended and 30 mm open-ended canals) was used to simulate various cavity dimensions. The block was submerged 3 mm deep in a basin of distilled water, and a conical fiber tip (PIPS 600 μm, Fotona) was positioned in the center of the cross section of the canal, 5 mm below the water surface, to deliver the Er:YAG laser pulses. A signal generator (SG; Tektronix, US, AFG 3102) was used to trigger the excitation laser and the camera.

**Fig. 5:**
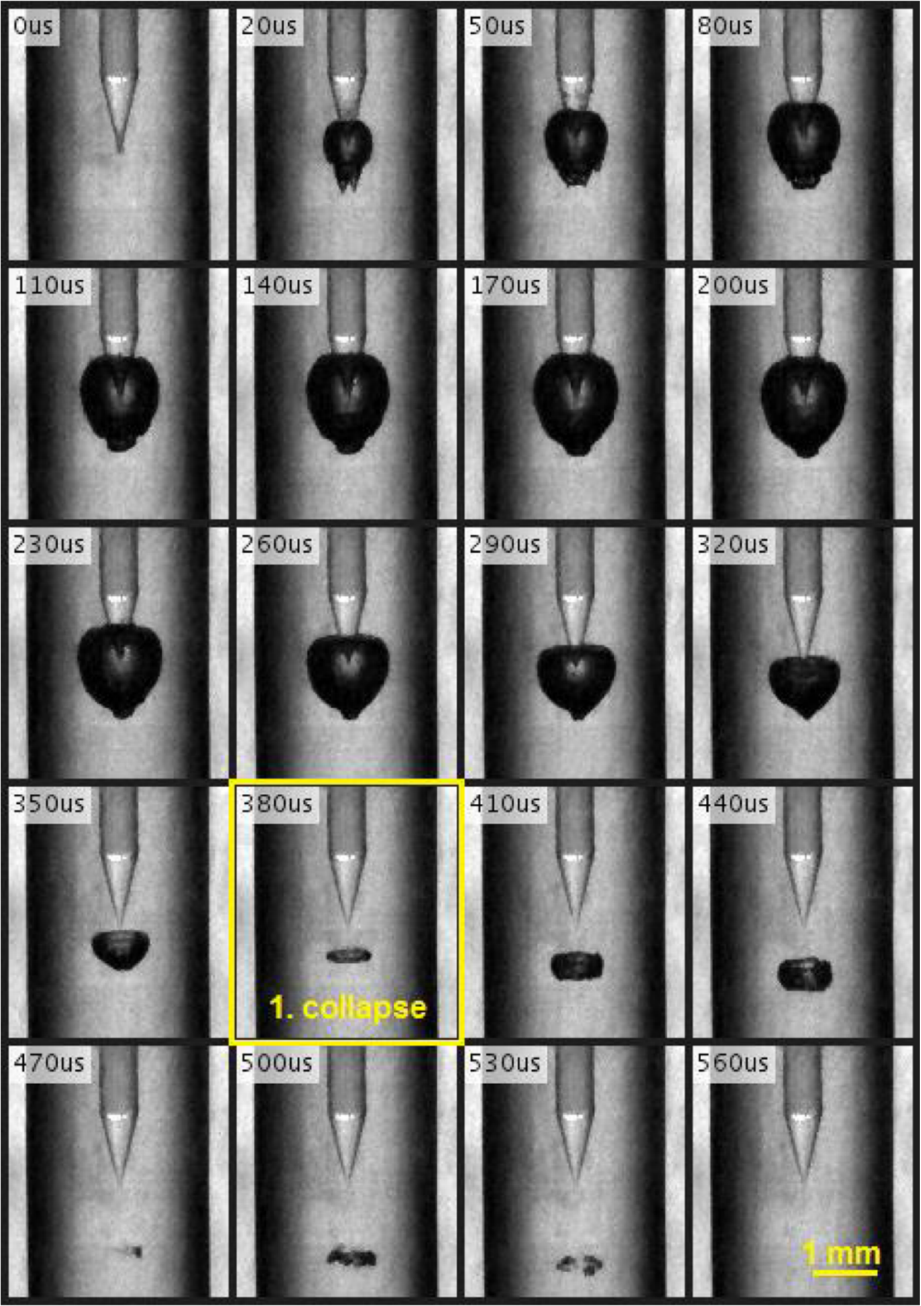
Typical sequence of a cavitation bubble’s oscillation, following an Er:YAG pulse with pulse energy *E*_p_ = 8 mJ and pulse width *t*_p_ = 50 μs, delivered through a conical fiber tip, in a 3-mm-diameter, 20-mm-long, closed-ended canal. The sequence was recorded using a high-speed camera at 100,000 frames per second and an exposure time of 250 ns. The first collapse of the bubble can be observed approximately 380 μs after the beginning of the laser pulse

Figure 5 shows a typical sequence of a cavitation bubble’s oscillation caused by a single 8 mJ laser pulse in a 20-mm-long, close-ended canal with a diameter of 3 mm. The bubble oscillation period *T*_OSC_ was measured as the time from the beginning of the growth of the cavitation bubble to its first collapse (marked by a yellow rectangle).

## Results

The first part of the experimental results demonstrates the amplification of pressure waves in confined canals when a second laser pulse is delivered at a proper delay. Since the required optimal delay depends on the first bubble’s oscillation period (*T*osc), we also measured the dependence of *T*osc on laser energy, cavity diameter and length, and on the position of the fiber tip within the cavity. In the second part, the presence of shockwaves during the first bubble’s collapse phase is demonstrated for the optimally synchronized laser pulse pair.

### Amplification of pressure waves

**Fig. 6:**
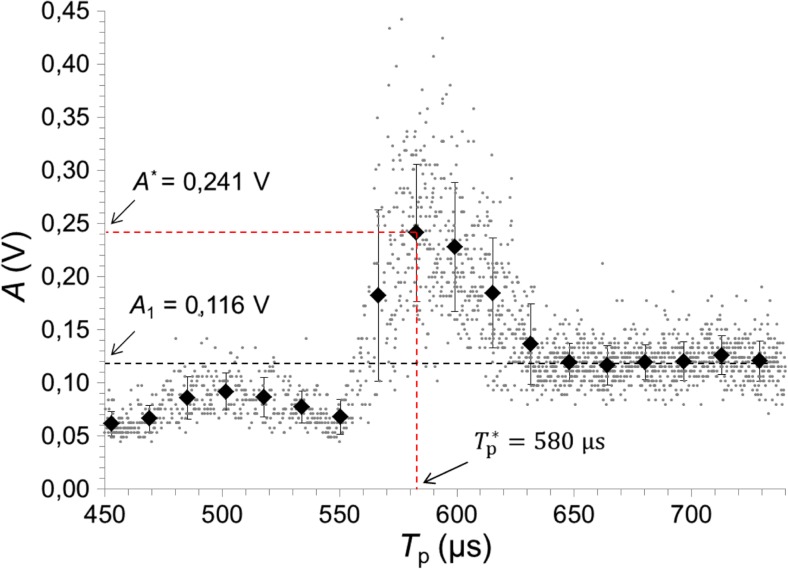
Amplitudes of the LBDP signal at the collapse of the bubble for various *T*_p_, ranging from 450 to 740 μs, in a 2-mm-diameter canal. Each dot represents a measurement of peak-to-peak amplitude of the pressure wave caused by the cavitation bubble’s collapse. The diamonds represent the means of grouped data with respective standard deviations. Er:YAG laser pulses with pulse energy *E*_p_ = 20 mJ and pulse width *t*_p_ = 50 μs were delivered through a flat fiber tip in this experiment

Figure 6 depicts the measured collapse amplitude (*A*) for various *T*_p_, ranging from 450 to 740 μs in a canal with a diameter *D* = 2 mm. In the case of a single laser pulse, the average (baseline) amplitude (*A*_1_) was 116 mV. For *T*_p_ bellow approximately 550 μs, the collapse amplitude is significantly diminished in comparison with what it would be in the absence of a second pulse. For *T*_p_ in the optimal range from 560 to 630 μs, the pressure waves are amplified. And for *T*_p_ longer than 630 μs, the collapse amplitude returns to the baseline level of a single laser pulse, since at longer delays, the second pulse is delivered after the collapse of the first oscillation bubble has already occurred.

The average maximal collapse amplitude at the optimal delay of ${T}_p^{\ast }$ = 583 μs was A^*^ = 241 mV, which is by a factor of 2.08 higher than *A*_1_. The optimal delay ${T}_p^{\ast }$ was determined as the midpoint of the class interval with the highest mean collapse amplitude.

**Fig. 7:**
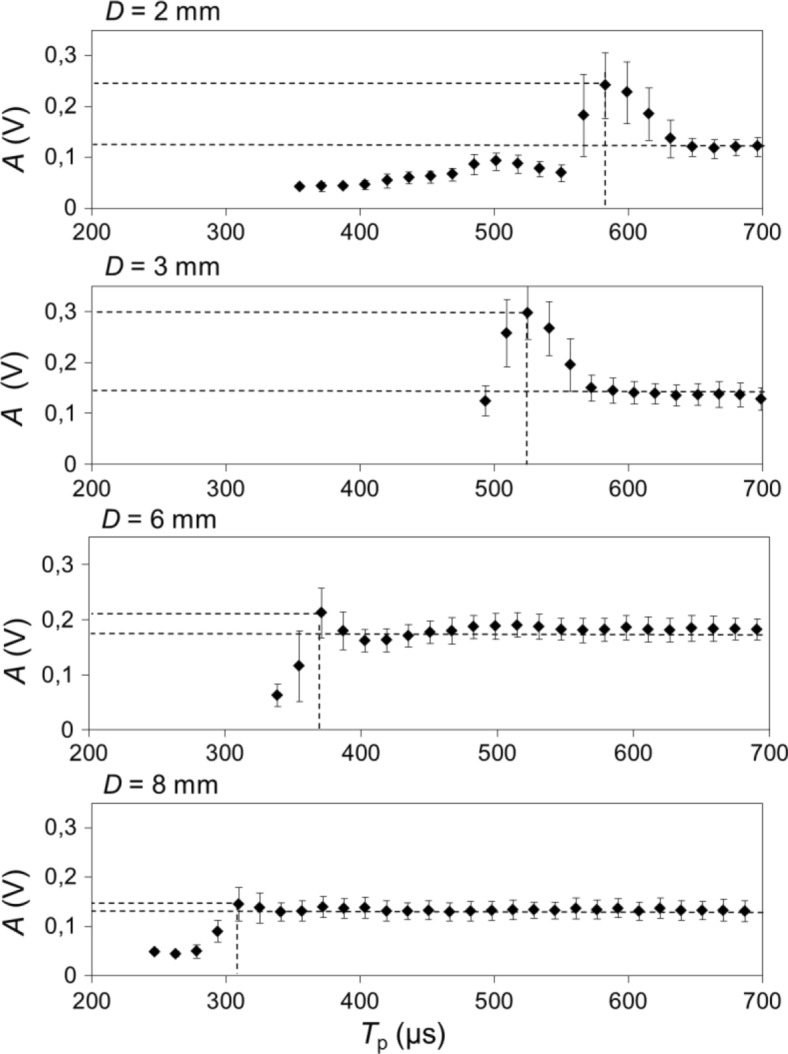
Measured collapse amplitudes A as a function of *T*_p_, for different canal diameters (D = 2, 3, 6 and 8 mm). Er:YAG laser pulses with pulse energies *E*_p_ = 20 mJ and pulse widths *t*_p_ = 50 were delivered through a flat fiber tip in this experiment

The dependence of the collapse amplitude A on *T*_p_ was measured also for other canal diameters (see Fig. 7). As can be seen from the obtained results, both A* and ${T}_p^{\ast }$ are strongly dependent on the canal dimensions. Generally, the ${T}_p^{\ast }$ and pressure wave amplification factor (*A*_f_ = A^*^/*A*_1_) decrease with the canal diameter. The optimal delay times and amplification factors for different canal diameters are collected in Table 1. The amplification of pressure waves (*A*_f_) is most pronounced in smaller diameter cavities, ranging from *A*_f_ = 1.09 for the *D* = 8 mm cavity to *A*_f_ = 2.2 and *A*_f_ = 2.08 for the *D* = 3 and 2 mm cavities, respectively. It is worth noting that shock waves are emitted at shock speeds close to the collapsing bubble but become considerably slower as they travel approximately 26 mm deep into the canal, where the measurement of the pressure waves was made. Therefore, it is expected that the actual amplification of the pressure waves in the vicinity of the collapsing bubble is much larger than shown in Table 1. This was confirmed also by our observation that when a standard conical PIPS fiber tip was used, the fiber’s cone became very quickly damaged when the optimal pulse separation was used.

**Table 1:** Optimal delay times and amplification factors for different diameter canals

Diameter (mm)	${T}_p^{\ast }$ (μs)	*A*_f_
2	580	2.08
3	525	2.20
6	370	1.16
8	310	1.09

**Fig. 8:**
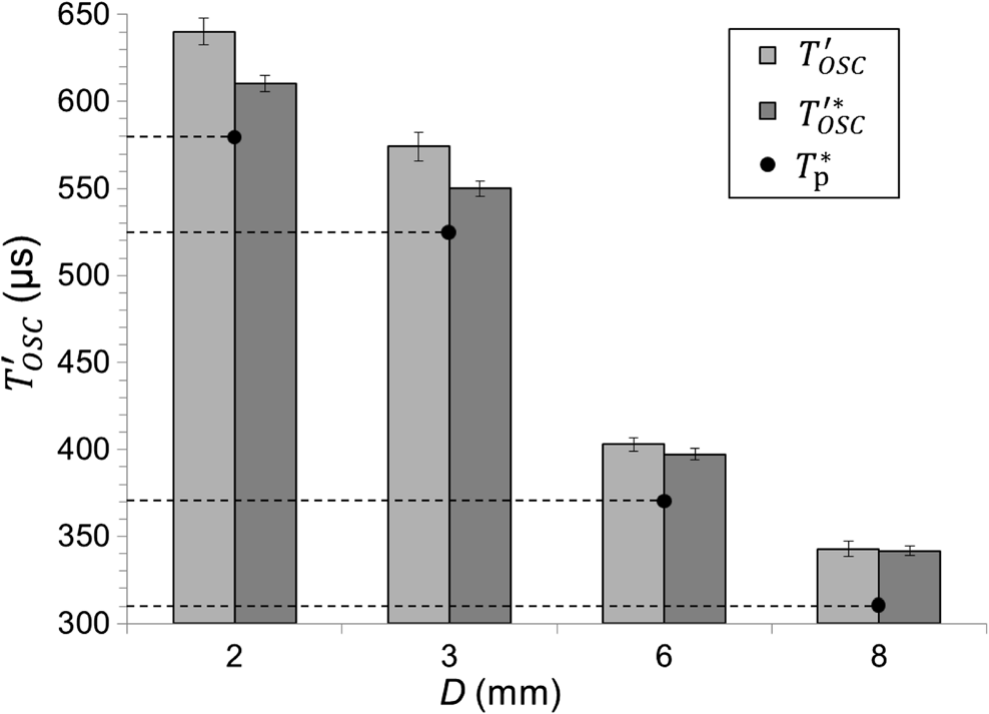
First bubble’s oscillation time as measured by the LBDP in the case of a single pulse (***T***′_**OSC**_) and in the case of two synchronized pulses (${\boldsymbol{T}}_{\mathbf{OSC}}^{\ast \prime }$) separated by ${\boldsymbol{T}}_{\mathbf{p}}^{\ast }$. Er:YAG laser pulses with pulse energy *E*_p_ = 20 mJ and pulse width *t*_p_ = 50 μs were delivered through a flat fiber tip in this experiment

Figure 8 shows, for different diameter canals, the difference in the first bubble’s LBDP oscillation times (*T*′_OSC_), both for cases when only a single laser pulse is emitted and for when the first pulse is followed by an optimally delayed second laser pulse. As can be seen, the optimally delayed second pulse (i.e., separated by T*_p_ from the first pulse) accelerates the first bubble’s collapse, resulting in a reduced LBDP oscillation time T*′_OSC_, The reduction ranges from 1 μs in the *D* = 8 mm diameter canal to 30 μs in the *D* = 2 mm diameter canal. The difference between the means of the first bubble LBDP oscillation times depending on whether a second laser pulse is present or not is significant at *P* < 0.001 for 2, 3, and 6 mm diameter canals and at *P* < 0.05 for the 8 mm diameter canal.

**Fig. 9:**
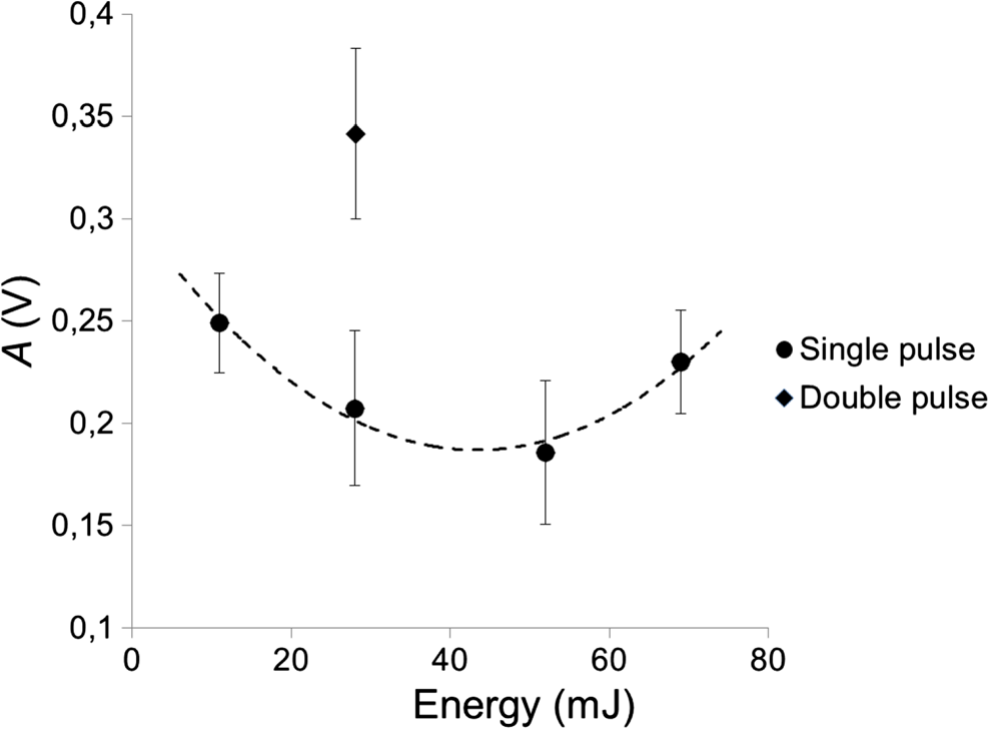
Measured collapse amplitude as a function of single-pulse (with pulse width *t*_p_ = 50 μs) laser energy (6 mm diameter canal, flat fiber tip)

Figure 9 shows the dependence of the first bubble’s collapse amplitude *A* on the first laser pulse’s energy. The pulse energy was controlled with a series of apertures of different diameters to keep the temporal profile of the laser pulse constant. The circles represent single-pulse results, and the diamond represents the collapse amplitude A for a case when the first laser pulse was followed by an optimally delayed second laser pulse with the same laser pulse energy. As can be seen from Fig. 9, increasing the individual laser pulse energy does not result in a significant increase in the collapse amplitude. In fact, the collapse amplitude gets even smaller when the laser energy is increased from 10 to 50 mJ, which we attribute to the increase of the bubble’s volume relative to the dimension of the canal. It is only when a second, optimally delayed laser pulse is added to the first pulse that the collapse amplitude of the first bubble gets significantly amplified.

### Dependence of the bubble oscillation period on experimental conditions

**Fig. 10:**
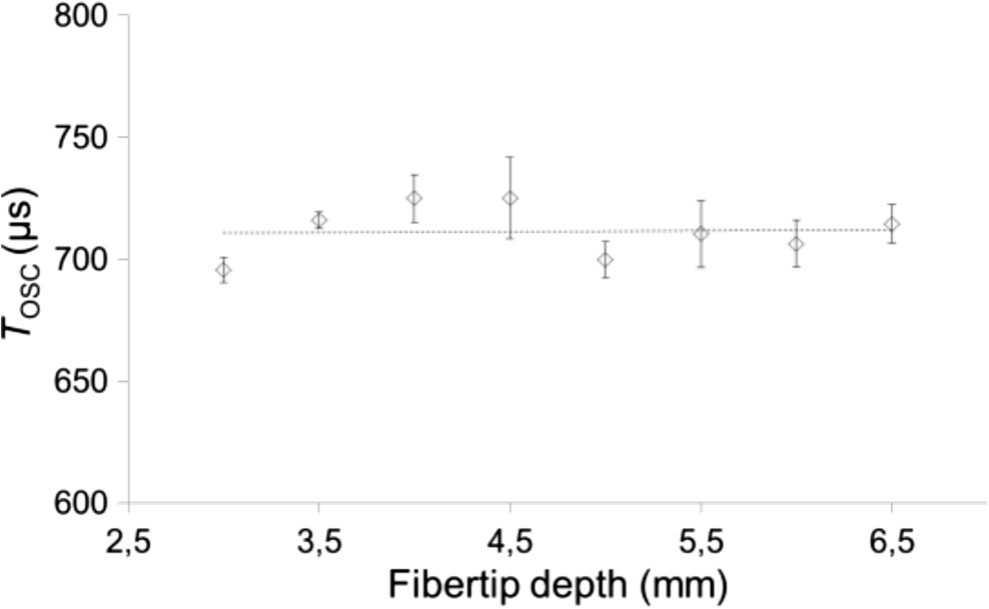
*T*_OSC_ as a function of the fiber tip depth inside a 10-mm-long canal with a diameter of 4 mm. Er:YAG laser pulses with pulse energy *E*_p_ = 30 mJ and pulse width *t*_p_ = 50 μs were delivered through a conical fiber tip in this experiment

The optimal separation of a synchronized laser pulse pair (*T*_p_) depends on the bubble oscillation period (*T*_OSC_) which further depends on specific experimental conditions. Figure 10 shows *T*_OSC_ as a function of the depth of the fiber tip inside a *L* = 10-mm-long open-ended canal with a diameter of *D* = 4 mm. The fiber tip depth represents the distance from the upper edge of the canal to the exit end of the fiber tip. For depths ranging from 3 to 6.5 mm, we observed no significant influence on *T*_OSC_. The small variations in *T*_OSC_ (ranging from 695 to 725 μs) can be attributed to slight differences in the radius alongside the canal and to the measurement error. The absolute depth of the fiber tip (distance from the water surface) in the measured range would only cause an increase in the hydrostatic pressure of approximately 0.35 mbar (representing the water hydrostatic pressure at the 6.5 mm depth), and therefore, any effect of the absolute depth on *T*_OSC_ is expected to be insignificant.

**Fig. 11:**
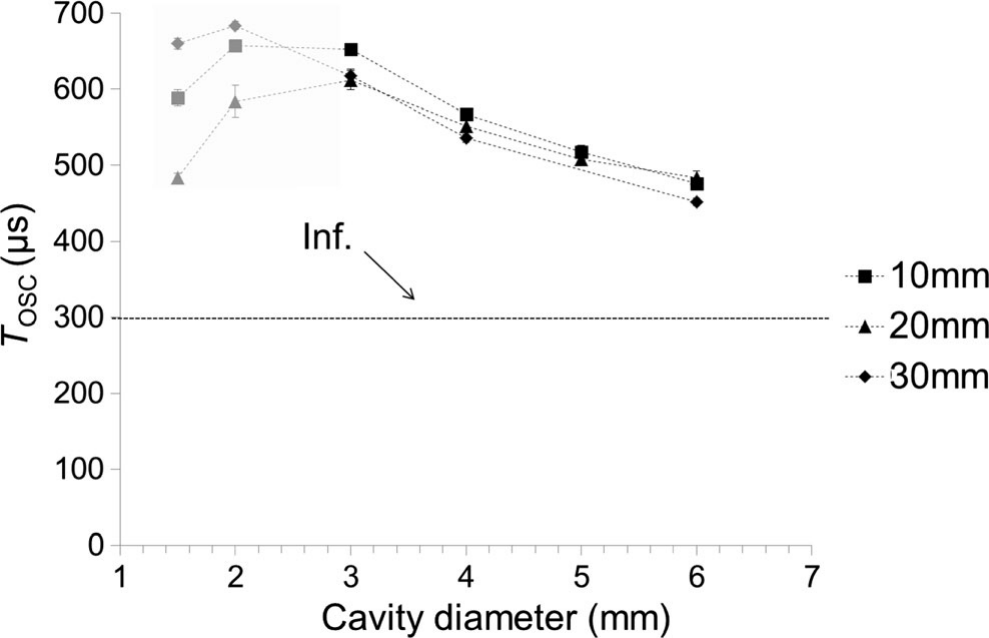
*T*_OSC_ as a function of the cavity diameter (ranging from 1.5 to 6 mm) for the close-ended canals of length 10 and 20 mm and for an open-ended 30 mm long canal. The dotted horizontal line represents *T*_OSC_ in an infinite reservoir. Er:YAG laser pulses with pulse energy *E*_p_ = 20 mJ and pulse width *t*_p_ = 50 μs were delivered through a conical fiber tip in this experiment.

Figure 11 shows *T*_OSC_ as a function of the cavity diameter (ranging from *D* = 1.5 to 6 mm) for close-ended cavities of different lengths (*L* = 10 and 20 mm) and in the case of an open-ended 30-mm-long canal. Results show that there is a strong negative correlation between the diameter of the canal and *T*_OSC_. At small canal diameters (2 and 1.5 mm), the cavitation bubble expands beyond the upper edge of the canal, which results in a shorter *T*_OSC._

**Fig. 12:**
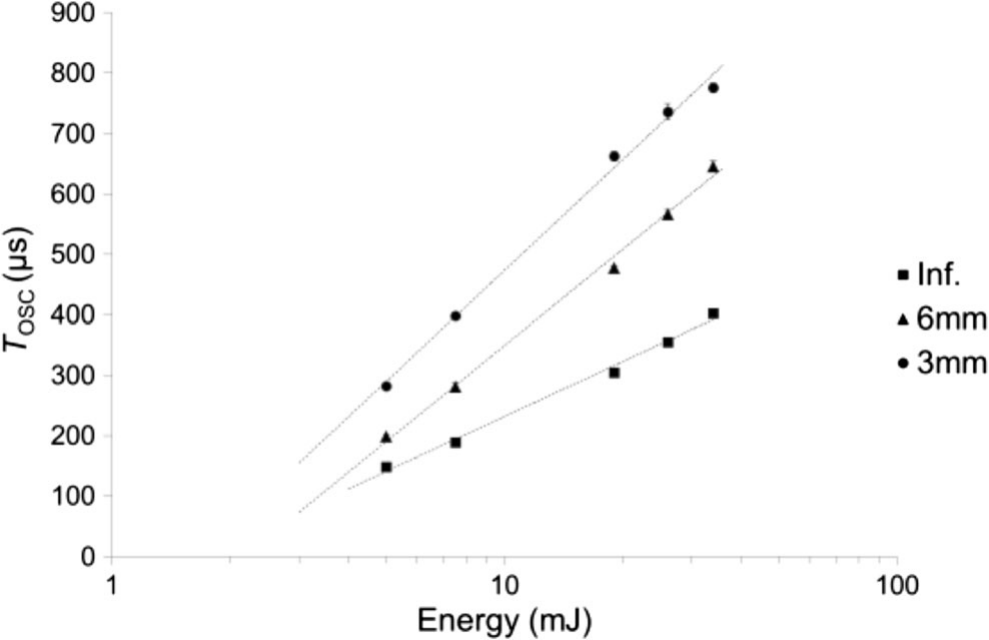
*T*_OSC_ as a function of laser pulse energy in 3 and 6 mm diameter closed-ended canals and in an infinite reservoir. Er:YAG laser pulses with pulse width *t*_p_ = 50 μs were delivered through a conical fiber tip in this experiment.

Figure 12 shows *T*_OSC_ as a function of laser pulse energy in 3 and 6 mm diameter closed-ended canals and in an infinite liquid. Results show that there is a strong positive correlation between the laser pulse energy and *T*_OSC_.

### Images of shockwaves generated during bubble collapse

**Fig. 13:**
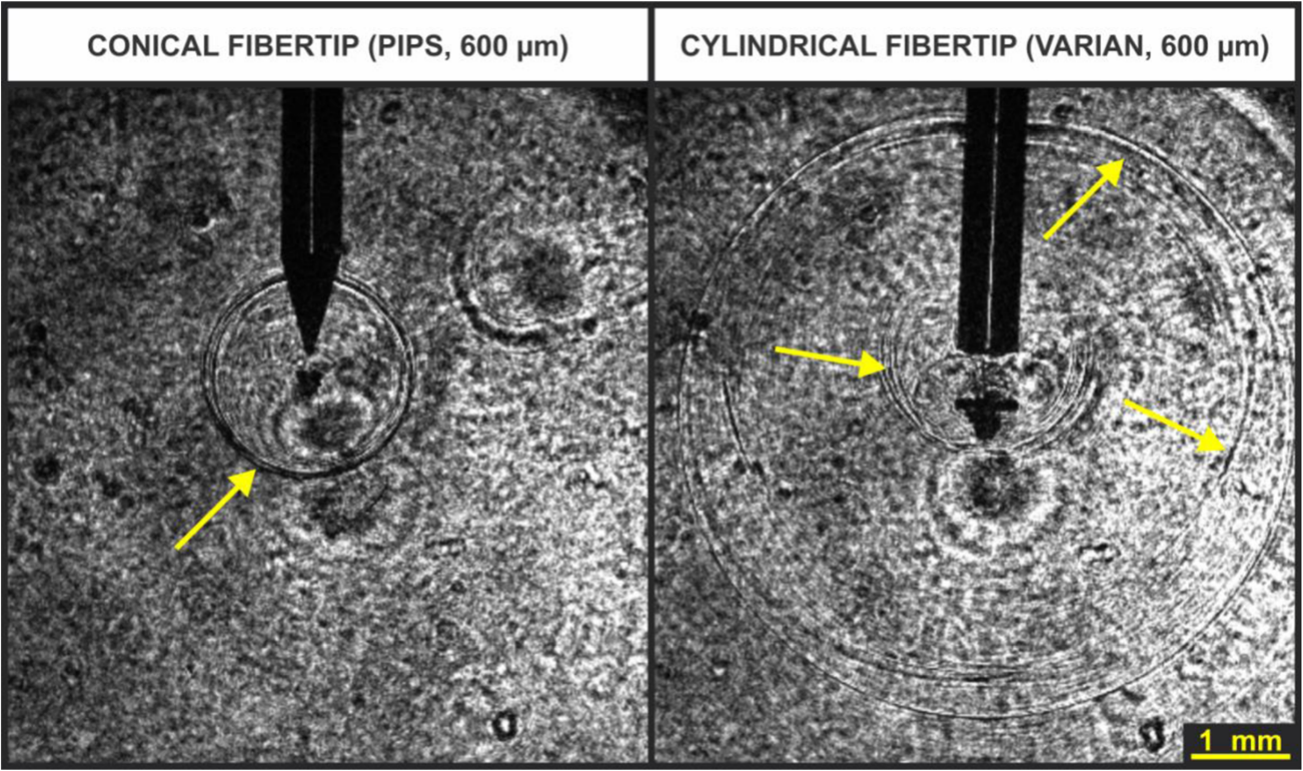
Typical images of shock waves recorded for a single laser pulse in an infinite liquid reservoir using shadow photography

Figure 13 shows typical shadow-graphic images of shockwaves as observed during the collapse of a single cavitation bubble in an infinite liquid reservoir. Since the shockwave causes a strong disturbance of water’s refractive index, it can be visualized as a sharp circular edge on the shadow-graphic images (yellow arrows are pointing to some of them). It is interesting to observe that multiple shockwaves are generated as a consequence of a divided bubble’s collapse. This is especially evident when a flat fiber tip is used.

**Fig. 14:**
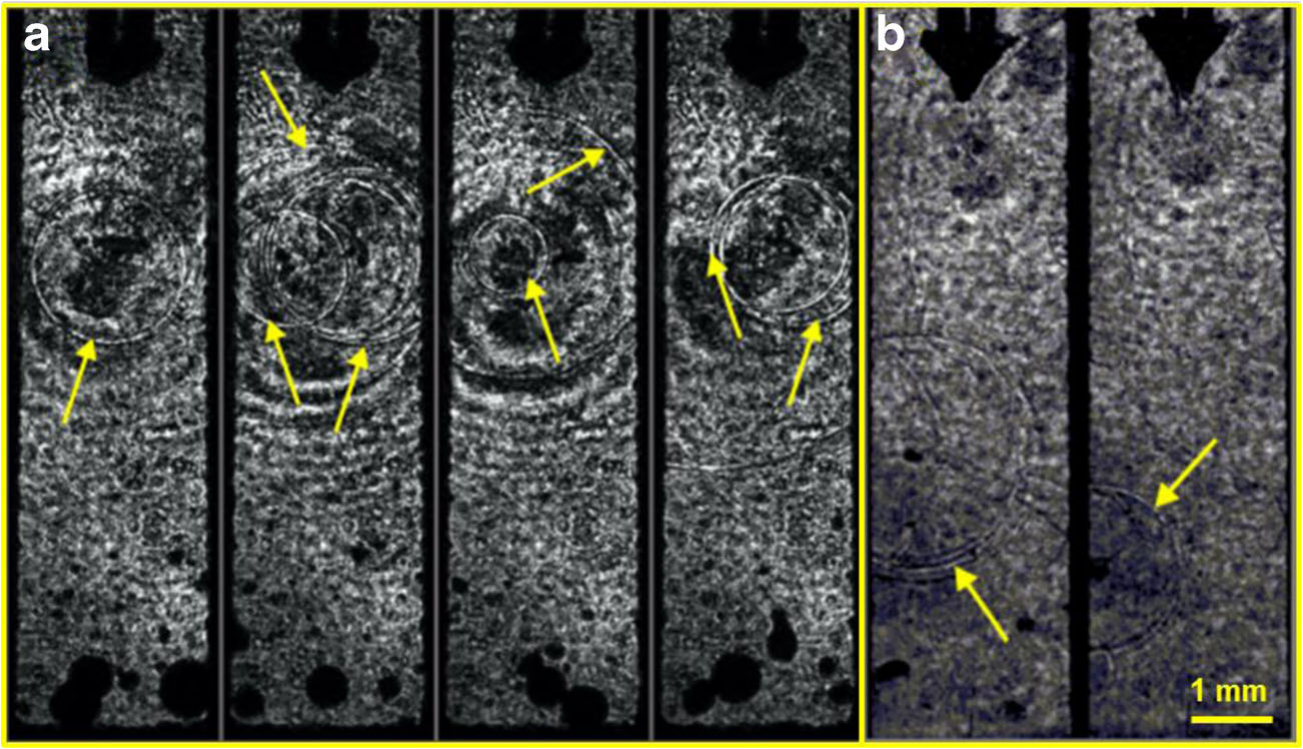
**a** Four examples of detected shock waves in a 3-mm-wide narrow canal as a result of the collapse of an initial bubble accelerated by the pressure exerted by the growing subsequent bubble. The beginning of the formation of the subsequent bubble can be seen at the bottom of the flat fiber tip. **b** Two examples of detected shock waves being emitted by the collapsing secondary bubbles. Er:YAG laser pulses with pulse energy *E*_p_ = 20 mJ and pulse width *t*_p_ = 50 μs were delivered through a flat fiber tip in this experiment

As opposed to a single bubble collapse in an infinite reservoir, no shock waves were observed during the collapse of a single cavitation bubble in spatially limited closed-ended canals, in agreement with previous reports [27]. However, when a subsequent laser pulse is emitted during the initial bubble’s collapse, the growth of the subsequent bubble exerts pressure on the collapsing initial bubble. This accelerates the collapse of the initial bubble and causes the emission of shock waves even in spatially limited water reservoirs. Figure 14a shows shadow-graphic images of shockwaves being emitted during the collapse of an initial cavitation bubble in a narrow canal. The beginning of a subsequent bubble expansion can be noticed on all images, which indicates that the collapse of the initial bubble was accelerated by a properly delayed subsequent laser pulse.

Smaller secondary bubbles are also formed alongside the entire canal. The violent collapse of the initial bubble also initiates the collapses of the secondary bubbles. Figure 14b shows the emission of shock waves from the collapsing secondary bubbles.

## Discussion

A major mechanism of action of currently used laser activated root canal irrigation techniques is believed to be the rapid fluid motion in the canal as a result of expansion and implosion of vapor bubbles, resulting in a more effective delivery of the irrigants throughout the complex root canal system [7, 15]. An additional mechanism which contributes to the efficacy of LAI is the improved removal of the smear layer, microorganisms, and biofilm as a result of the physical action of the turbulent irrigant [7, 15]. In addition, chemical action seems to play a role as well [18, 28]. For example, an increased reaction rate of NaOCl was found upon activation by a pulsed erbium laser [28]. By being able to generate shock waves within narrow root canals, we hypothesize that both the physical and chemical actions of LAI can be further enhanced by using the SWEEPS technique.

Experimental results of the SWEEPS technique show that significant amplification of pressure waves can be achieved with optimal delay times of the second laser pulses (see Fig. 7 and Table 1). It is important to note that the amplitude of collapse is significantly higher if a double-pulse regime is used compared to a single-pulse with the same cumulative energy (see Fig. 9), because increased single-pulse energy leads to an increase in the volume of the cavitation bubble relative to the cavity dimensions, which in turn leads to a weakened collapse. The main mechanism of this amplification is in our opinion the acceleration of the initial bubble collapse, which is significantly diminished in confined spaces (like root canals).

This hypothesis is confirmed by the results shown in Fig. 8, where oscillation time as measured by the LBDP in the case of a single pulse (*T*′_OSC_) and in the case of a synchronized pulse pair ($T{\prime}_{\mathrm{OSC}}^{\ast }$) is shown. Slight differences between *T*′_OSC_ and $T{\prime}_{\mathrm{OSC}}^{\ast }$ could be explained by the increased speed of propagation of the pressure waves. However, the distance between the source of the pressure wave and the probe laser beam is approximately 25 mm, which means a travel time of roughly 17 μs at the speed of sound in water [29, 30]. Therefore, the increased speed of propagation cannot account for the 30 μs (see Fig. 8, 2-mm-diameter canal) difference between *T*′_OSC_ and $T{\prime}_{\mathrm{OSC}}^{\ast }$. Furthermore, since shock waves traveling at supersonic speed quickly converge towards the speed of sound [31, 32], we do not expect a significant effect on the average speed of propagation of the pressure wave over a relatively great distance (25 mm). Similarly, slight differences in *T*′_OSC_ could be the result of the collapse of the bubble happening closer to the probe laser beam, perhaps being pushed downward by the expanding second bubble. High-speed camera observations confirm that this effect is not large enough to contribute significantly to the difference between *T*′_OSC_ and ${T}_{\mathrm{OSC}}^{\prime \ast }$. However, the differences between *T*′_OSC_ and $T{\prime}_{\mathrm{OSC}}^{\ast }$ are consistent with the collapse happening earlier due to the exerted pressure of the second expanding bubble on the collapsing bubble, accelerating the collapse.

The acceleration factor (*A*_cc_) was defined as an increase in the average speed of collapse after the initiation of the second pulse:

$$ {A}_{\mathrm{cc}}=\frac{T{\prime}_{\mathrm{OSC}}-{T}_{\mathrm{p}}^{\ast }}{T{\prime}_{\mathrm{OSC}}^{\ast }-{T}_{\mathrm{p}}^{\ast }} $$

**Fig. 15:**
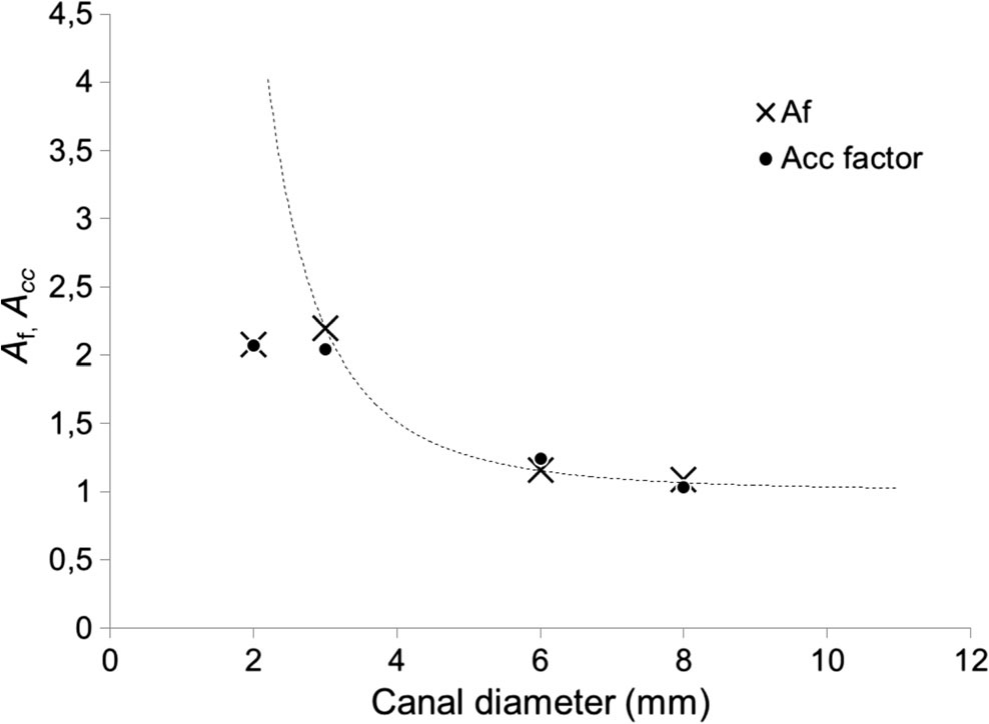
Amplification factor (*A*_f_) and acceleration factor (*A*_cc_) in canals of various diameters. The dotted line is a visual aid only and represents a power function fit to the 3–8 mm canal data

The strong covariance between *A*_f_ and *A*_cc_ for various canal diameters, which is shown in Fig. 15, supports the hypothesis that the amplification of the shock waves is a result of the acceleration of the collapse of the bubble. The results of *A*_f_ and *A*_cc_ for the 2-mm-diameter canal are consistent with measurements of the actual bubble oscillation period *T*_OSC_ (see Fig. 11) and are likely caused by the cavitation bubble partially extending outside the boundaries of the canal during its growth, changing the observed dynamics.

It is important to note that the enhanced emission of shock waves does not appear to result in an increased apical irrigant extrusion. Recently, a study of the potential apical irrigant extrusion during the SWEEPS laser irrigation was carried out [33], during which irrigation using two standard endodontic irrigation needles (notched open-end and side-vented) was compared with the PIPS and SWEEPS laser irrigation procedures. Both the PIPS and SWEEPS irrigation procedures resulted in a significantly lower apical extrusion compared to the conventional irrigation with endodontic irrigation needles, in agreement with a previous report [34].

Finally, in our experiments, the single pulses or pairs of pulses were delivered at low repetition rates of up to 0.2 Hz. A potential dependence of the SWEEPS phenomena on the increased pulse pair repetition rate was not explored.

## Conclusion

A laser beam deflection probe, a high-speed camera, and shadow photography were used to characterize the effects of synchronized delivery of Er:YAG pulses in a confined volume of water. As opposed to in infinite liquid reservoirs, shock waves are typically not emitted by laser-induced cavitation bubbles in confined liquid spaces. This limits the surface cleaning efficacy of the laser-induced cavitation bubbles. However, as our study shows, pressure waves caused by the collapse of a laser-induced cavitation bubble can be significantly amplified (*P* < 0,001) also in a confined reservoir. This is achieved by delivering a subsequent laser pulse, separated from the initial pulse by a proper temporal delay. It is to be noted that similar amplification cannot be achieved by simply increasing the laser pulse energy. Larger single-pulse energies lead to larger cavitation bubbles relative to the cavity dimensions, which in turn results in a weakened collapse of the bubbles. On the other hand, applying a subsequent laser pulse during the initial bubble’s collapse leads to the growth of a second bubble, which exerts pressure on the collapsing initial bubble, accelerating its collapse and causing the emission of shock waves. Results show that the optimal delay between the two laser pulses is strongly correlated with the cavitation bubble’s oscillation period. The resulting amplification is most pronounced in smaller diameter canals (< 3 mm). Measurements with a high-speed camera show that the oscillation periods of cavitation bubbles depend strongly on laser pulse energy and canal diameter, as opposed to the canal length and fiber tip depth, which have only a minor influence on the bubbles’ oscillation period.

The observed shock wave-enhanced emission photo-acoustic streaming (SWEEPS) phenomenon could be used to improve the efficacy of laser-assisted root canal treatment, especially with respect to the smear layer and biofilm removal. Because of the variability of root canal geometries, further methods of improvement may be needed in order to achieve a reliable synchronization between the bubble oscillation and the laser pulse pair timing. One potential improvement may be a special laser modality in which the temporal separation between the pairs of laser pulses is continuously swept back and forth in order to ensure that during each sweeping cycle the optimal separation between the pulse pair is achieved, as required for shock wave generation [35].
